# Current status and recent advances in reirradiation of glioblastoma

**DOI:** 10.1186/s13014-021-01767-9

**Published:** 2021-02-18

**Authors:** Giuseppe Minniti, Maximilian Niyazi, Filippo Alongi, Piera Navarria, Claus Belka

**Affiliations:** 1 Department of Medicine, Surgery and Neurosciences, University of Siena, Policlinico le Scotte, 53100 Siena, Italy; 2 IRCCS Neuromed, Pozzilli, IS Italy; 3 Department of Radiation Oncology, University Hospital, LMU Munich, Munich, Germany; 4 German Cancer Consortium (DKTK), Partner Site Munich, Munich, Germany; 5 Advanced Radiation Oncology Department, Cancer Care Center, IRCCS Sacro Cuore Don Calabria Hospital, Negrar, VR Italy; 6 Radiotherapy and Radiosurgery Department, Humanitas Clinical and Research Hospital-IRCCS, Rozzano, MI Italy

**Keywords:** Target delineation, Recurrent glioblastoma, Reirradiation, Stereotactic radiosurgery, Hypofractionated radiotherapy, Radionecrosis

## Abstract

Despite aggressive management consisting of maximal safe surgical resection followed by external beam radiation therapy (60 Gy/30 fractions) with concomitant and adjuvant temozolomide, approximately 90% of WHO grade IV gliomas (glioblastomas, GBM) will recur locally within 2 years. For patients with recurrent GBM, no standard of care exists. Thanks to the continuous improvement in radiation science and technology, reirradiation has emerged as feasible approach for patients with brain tumors. Using stereotactic radiosurgery (SRS) or stereotactic radiotherapy (SRT), either hypofractionated or conventionally fractionated schedules, several studies have suggested survival benefits following reirradiation of patients with recurrent GBM; however, there are still questions to be answered about the efficacy and toxicity associated with a second course of radiation. We provide a clinical overview on current status and recent advances in reirradiation of GBM, addressing relevant clinical questions such as the appropriate patient selection and radiation technique, optimal dose fractionation, reirradiation tolerance of the brain and the risk of radiation necrosis.

## Introduction

Glioblastoma (GBM) is the most common malignant brain tumor in adults. The standard treatment includes surgery followed by external beam radiation therapy (RT) with concomitant and maintenance temozolomide, with a reported median survival time of 14.6 months and 2-year survival rate of 26.5%, respectively [1]. However, almost all GBMs relapse within or in close proximity to the initial site of disease despite advances in surgery and chemoradiotherapy.

For patients with a recurrent GBM, no standard of care exists. Treatment options include repeated surgery, reirradiation, systemic therapy, and best supportive care [2]. A surgical approach can be utilized for locally recurrent or progressive malignant glioma with reported median survival times ranging from 6 to 17 months, with total or subtotal (extent of resection > 80%) resection being associated with longer survival [3, 4]; however, factors associated with increased postoperative morbidity, e.g. location of tumor recurrence in eloquent/critical brain regions, low performance status, and tumor volume have to be carefully evaluated. For patients who receive salvage systemic treatment at recurrence, the antiangiogenic agent bevacizumab and alkylating agents, either temozolomide or lomustine, represent common salvage therapeutic options resulting in median survival times of 6 to 12 months [5–7]. Bevacizumab has been the standard salvage therapeutic option for patients with recurrent GBM in the United States since its approval in 2009 by the Food and Drug Administration [5, 6]; in contrast, lomustine is the recommended second-line chemotherapy in the European Union based on a large randomized trial of 437 patients with progressive GBM that showed a similar median survival time around 9 months for those receiving lomustine plus bevacizumab versus lomustine alone [7].

Reirradiation is increasingly used in patients with recurrent GBM. Even though damage of normal brain tissue previously treated with high dose RT is of concern, technological advances in radiation techniques, including developments in treatment planning systems and dose delivery, have improved the therapeutic ratio making it possible to use reirradiation as feasible treatment option [8]. Variable median survival times of 6 to 12 months and neurological toxicity rates of 5% to 20% have been reported after stereotactic radiosurgery (SRS) and stereotactic radiotherapy (SRT), using either hypofractionated or conventionally fractionated radiation schedules [9, 10]. In addition, survival benefit has been reported following reirradiation in combination with temozolomide or bevacizumab compared to reirradiation alone. We present a clinical overview on the current status and advances of reirradiation in the setting of recurrent or progressive GBM after standard treatment, with special regard to target volume delineation and impact of radiation techniques on survival and risk of radiation-induced brain necrosis.

## Imaging and delineation of target volumes

A second course of radiation remains of concern for patients with a recurrent GBM because of unacceptable risk of neurological toxicity in the form of radiation necrosis. Then, the key issue for GBM reirradiation is an accurate delineation of target volumes and organs at risk (OARs) for a precise calculation of the spatial dose distribution, and for choosing the optimal radiation dose fractionation schedule. Magnetic resonance imaging (MRI), using contrast-enhanced T1-weighted and T2-weighted sequences, are routinely used because of their more accurate depiction of the extension of the tumor and brain anatomy compared to computed tomography (CT). The gross tumor volume (GTV) is generally defined as the visible lesion on MRI contrast-enhanced T1-weighted sequences. The clinical target volume (CTV) which includes areas of potential suspected microscopic tumor infiltration and potential paths of microscopic spread, is then generated by adding a variable margin of 0–5 mm to the GTV constrained at anatomical borders, e.g. tentorium, falx cerebri, and bone. In this regard, no GTV-to-CTV margins are usually utilized during SRS with the aim to limit the risk of toxicity, where margins up to 5 mm are commonly applied during hypofractionated and conventionally fractionated SRT (see below). In a few studies, CTV delineation consists of including FLAIR abnormalities around contrast-enhancing lesion in T1-weighted sequences [11, 12]. Finally, depending on radiation technique, available technology, and institutional practice, an expansion up to 3 mm is applied to generate the planning target volume (PTV) which accounts for uncertainties in treatment planning and delivery.

Positron emission tomotherapy (PET)/CT imaging with radiolabeled amino acids may help to improve target volume delineation accuracy by revealing tumor infiltration in regions with a non-specific MRI appearance [13–18]. In a trial including 44 patients with recurrent high-grade gliomas who received reirradiation with hypofractionated SRT (30 Gy/6 fractions) with or without temozolomide, Grosu et al. [13] demonstrated significantly longer median survival time using treatment planning based on (11)C-methionine (MET)-PET or (123)I-alpha-methyl-tyrosine (IMT) single-photon computed emission tomography/CT/MRI image fusion compared with treatment planning using CT/MRI alone (9 vs. 5 months; p = 0.03). Significantly improved survival using MET-PET/CT to define the GTV compared with treatment planning based on conventional MRI has been observed by others [14]. In a prospective imaging study comparing FET-PET with advanced MRI imaging in 41 patients who received SRT for recurrent GBM, Popp et al. [17] found that target volume delineation using MET-PET imaging correlated better with localization of post-reirradiation recurrences in comparison to target volume delineation based on diffusion-weighted (DWI) MRI and apparent diffusion coefficient (ADC) maps which reveal regions of high cellularity as surrogate for active tumor. Currently, a multicenter phase II trial (GLIAA, NOA-10, ARO 2013/1) is seeking to evaluate whether reirradiation planning using FET/PET improves clinical outcome in patients with recurrent GBM compared to contrast-enhanced MRI [19]. Although these studies support the use of biologic imaging as an effective strategy for target delineation of recurrent GBM, the impact of PET-based treatment planning on survival requires further investigation.

## Brain tolerance to reirradiation

Normal brain tissue dose tolerance is the limiting factor when giving reirradiation. An estimated risk of symptomatic radiation necrosis has been determined following brain SRS and SRT [20, 21]. For conventional fractionation, a 5% and 10% risk of symptomatic radiation necrosis is predicted to occur at a biologically equivalent dose (BED) of 72 Gy (range, 60–84 Gy) and 90 Gy (range, 84–102 Gy) in 2-Gy fractions. For SRS, the risk of complications increases rapidly once the volume of the brain exposed to 12 Gy is more than 5–10 ml [22, 23]. Dose-volume predictors of toxicity for critical structures, e.g. optic chiasm and brainstem, have also been determined for SRS and SRT, both hypofractionation and normofractionation [24–26]; however, high-level evidence is lacking and current constraints should be used with caution.

When assessing the risk of radiation necrosis following reirradiation, several factors should be taken into account, including dose and fractionation, treated volume, combined chemotherapy, and interval between radiation treatments. Previous meta-analyses of brain reirradiation found no cases of brain necrosis when the cumulative radiation dose of the two courses of radiation, calculated as biological equivalent total dose normalized to 2 Gy/fraction (EQD2) using the linear-quadratic model, was < 96 Gy [27, 28]. The median cumulative EQD2 reported in conventionally fractionated reirradiation (81.6–101.9 Gy) series was generally lower than those observed in hypofractionated SRT (90–133.9 Gy) and SRS (111.6–137.2 Gy). The estimated risk of radiation necrosis at 1 year was 2–12% for cumulative EQD2 > 96.2 Gy and up to 17% for cumulative EQD2 > 137 Gy. Our update of the literature on GBM reirradiation confirms the relationship between cumulative EQD2 and the risk of radiation necrosis, as shown in Tables 1, 2 and 3; the reported risk was about 0–3% after conventional fractionation at cumulative EQD2 < 101 Gy, 7–13% after hypofractionated SRT at cumulative EQD2 of 102–130 Gy, and up to 24.4% after SRS using a cumulative EQD2 of about 124–150 Gy.

**Table 1:** Stereotactic radiosurgery in recurrent patients with glioblastoma

Author	Pts (No)	Interval between RT courses	SRS modality	Median dose (Gy)	Imaging for planning	Treated volume (ml)	CTV/PTV margins (mm)	Systemic therapy	Median PFS (months)	Maedian OS (months)	EQD2 (Gy)	Cumulative EQD2 (Gy)	RN (%)
Larson et al., 2002 [35]	53	14.5	GK	16 15 (+ M)	T1-w ce MRI	SRS + M, 8 SRS, 9.1	NR	M, 12	4.2	4.4 9.5 (+ M)	72 63.8 (+ M)	132 123.8 (+ M)	8
Combs et al., 2005 [36]	32	10	LINAC	15 (10–20)	T1-w ce MRI	10 (1.2–59.2)	2–5	None	5	10 38% at 1 yr	63.8	117.8	0
Kong et al., 2008 [37]	65	4.3	GK	16 Gy	T1-w ce MRI	10.6 (0.1–79.6)	None	None	4.6	13 58.4% at 1 yr	72	132	24.4
Cuneo et al., 2009 [38]	49	20	LINAC	15	T1-w ce MRI, Some PET/CT	4.8	0–1	BEV	5.2 (+ BEV) 2.1 (-BEV)	11.9 (+ BEV) 3 (-BEV)	63.8	123.8	10
Patel et al., 2009 [39]	26	12.5	LINAC	18 (12–20)	T1-w ce MRI	10.4	None	CCNU, TMZ	NR	8.4	90	150	4
Pouratian et al., 2009 [40]	26	NR	GK	17	T1-w ce MRI	21.3	None	None	NR	9.4	80.7	140.7	0
Skeie et al., 2012 [41]	77	8.9	GK	12.2	T1-w ce MRI	12.4	NR	PCV	6	12	36.2	96.2	9.8
Dodoo et al., 2014 [42]	35	NR	GK	20 (14–22)	T1-w ce MRI	4.8 (0.03 − 38.1)	NR	NR	NR	11.3	110	170	23
Martinez-Carrillo et al., 2014 [43]	46	10	LINAC	18 (14–20)	T1-w ce MRI	4 (0.05–34.1)	0–5	NR	NR	7.5	90	150	10
Pinzi et al., 2015 [44]	88	15	CK	16–22	T1-w ce MRI	2 (0.14–83)	0–1	CHT, 22 (type not specified)	NR	11.5 48% at 1 yr	63.8	123.8	6
Bokstein et al., 2016 45]	33	18	GK	18 (14–24)	T1-w ce MRI	2.2 (0.2–9.5)	NR	BEV, 6 TMZ, 15	5 (1.0–96.4)	15.9	90	150	5.5
Frisher et ai., 2016 [46]	42	17	GK	10	T1-w ce MRI	5.1	NR	TMZ, 28	4.4	9.6	30	90	2.4
Imber et al., 2017 [47]	174	8.7	GK	16 (10–22)	T1-w ce MRI	7.0 (0.3–39.0)	NR	TMZ, 20 CCNU, 13 BCNU, 11	NR	10.6	72	132	13
Kim et al., 2017 [48]	57	NR	GK	15	T1-w ce MRI	SRS + TMZ, 9.8; SRS, 11	NR	TMZ, 28	3.6 6 (+ TMZ)	9.2 15.5 (+ TMZ)	63.8	123.8	24.4
Sharma et al., 2018 [49]	53	16	GK	18 (12–24)	T1-w ce MRI	3.8 (0.01–29.7)	None	None	4.4	11.0	90	150	4
Morris et al., 2019 [50]	45	13.5	GK	17 (13–24)	T1-w ce MRI	2.2 (0.1–25.2)	None	BEV	5.2	13.3	80.7	140.7	0

SRS, radiosurgery; CTV, clinical target volume; PTV, planning target volume; PFS, progression-free survival; OS, overall survival; EQD2, equivalent dose normalized to 2 Gy;

NR, not reported; SRS, stereotactic radiosurgery; LINAC, linear accelerator; GK, Gamma Knife; T1-w ce MRI, T1-weighted contrast-enhanced magnetic resonance imaging;

TMZ, temozolomide; BEV, bevacizumab; M, marimastat; CCNU, lomustine; TMZ, temozolomide; BEV, bevacizumab

Although the validity of the linear-quadratic model has been questioned for high radiation doses as employed during SRS and ultrahypofractionated schedules [29], current data indicates that doses exceeding 100 Gy should be used with caution, especially in case of large irradiated volumes. In contrast, other factors did not appear to be linked with an increased risk of radiation necrosis, e.g. the time interval between two radiation courses or the use of concurrent bevacizumab, the latest associated with reduced treatment toxicity [30].

Based on dose/volume data and clinical risk estimates for central nervous system (CNS), maximum doses exceeding 8–10 Gy and 12 Gy given in single fraction and 55 Gy and 54 Gy (59 Gy to < 10 cc) given in 1.8–2 Gy fractions for optic apparatus and brainstem, respectively, should be avoided in clinical practice [26]; however, limited data are available in the setting of reirradiation. Niyazi et al. [31] showed no relevant long-term toxicity in a series of 58 patients who received reirradiation for a malignant glioma using maximum cumulative EQD2 of 80.3 Gy, 79.4 Gy, and 95.2 Gy to optic chiasm, optic nerves, and brainstem, respectively. With regard to the brainstem, a few series did not observe significant neurological toxicity and/or radiological changes suggestive of brain necrosis in patients with progressive diffuse intrinsic pontine gliomas receiving a second course of SRT with doses of 20–24 Gy given in 2-Gy fractions [32]. Overall, these data suggest relatively high and fast recovery capacity of the normal human brain after a second course of RT, similarly to that seen for spine [33], and support the use of cumulative EQD2 doses around 100 Gy or even higher (up to 120 Gy), e.g. small and well defined lesions away from eloquent areas.

Few data are available on the effect of reirradiation on cognitive deterioration. Wick et al. [34] reported the results of a phase II study involving 91 patients with progressive GBM randomized to receive reirradiation (36 Gy in 2-Gy fractions) with or without the systemic agent Asunercept. Neurological status and quality of life, assessed by the European Organization for Research and Treatment of Cancer (EORTC) Quality of Life Questionnaire (QLQ)-C15 PAL fatigue scale, EORTC QLQ-BN20 and Medical Research Council (MRC)-Neurological status, remained stable in both groups until progression. In another small prospective series of 15 patients who received hypofractionated SRT (35 Gy in 7-Gy fractions) for malignant gliomas, overall quality of life, physical functioning, and cognitive functioning remained stable in two thirds of the patients at a median time of 9 months. Although these results offer some reassurance about the safety of reirradiation in adult patients, caution should be applied in clinical practice when treating large volumes with large fraction size.

## Survival outcomes and toxicity

### SRS

For patients with recurrent GBM, SRS is usually given alone or in combination with systemic therapy. Treatment planning characteristics and clinical outcomes of sixteen selected series published from 2005 to 2020 and including 901 patients who received SRS for recurrent GBM are shown in Table 1 [35–50]. With a median dose of 15–18 Gy for a treated volume around 4–10 ml as seen in the majority of studies, the overall survival time from the date of SRS reirradiation ranges from 7.5 to 13 months and progression-free survival time from 4.4 to 6 months. Gamma Knife remains the prevalent SRS modality, whereas hypofractionated treatments are typically delivered with Cyberknife and LINAC. Of note, a recent systematic review and meta-analysis of 50 non-comparative studies including 2095 patients treated with different SRS reirradiation modalities/technologies showed similar overall survival rates of 70% and 34% and progression-free survival rates of 40% and 16%, respectively, at 6 and 12 months [10].

With respect to the retrospective nature and small numbers of patients, available data suggest increased survival rates with SRS and systemic therapies compared to SRS alone [38, 44, 46, 48, 51]. The reported overall survival time after concurrent SRS and temozolomide is around 9–15 months; some [44, 46, 48, 51], but not all [36, 47], studies showed significant survival benefit following chemoradiation over SRS alone, especially in patients with O^6^-methylguanine-DNA methyltransferase (MGMT) gene promoter methylation. Since its approval by the US Food and Drug Administration for the treatment of patients with recurrent GBM in 2009, the efficacy of the anti-vascular endothelial growth factor (VEGF)-A humanized monoclonal antibody bevacizumab in combination with SRS has been evaluated in several studies [38, 50, 52]. In a series of 49 patients with recurrent GBM who received SRS with or without concurrent and adjuvant bevacizumab, Cuneo et al. [38] observed median progression-free survival and overall survival times from SRS of 6 months and 10 months, respectively. The 1-year overall survival time was 50% for patients who received SRS and bevacizumab and 22% for patients receiving SRS alone (p = 0.005), with respective median progression-free survival times of 5.2 months and 2.1 months (p = 0.014). The superiority of combined treatment versus SRS alone has been seen in other small retrospective series [50, 52]; of note, the safety of SRS plus bevacizumab has been confirmed in several studies, with patients receiving bevacizumab who were less likely to develop grade III adverse radiation effects.

As shown in Table 1, the reported risk of symptomatic radiation necrosis for sixteen studies including 928 patients varies from 0 to 24.4%, being mainly related to radiation dose and treated volume. Using the cumulative EQD2 as predictor of the risk, we found that values around 120 Gy were generally associated with a risk < 10% for a median tumor volume of approximately 10 ml, whereas a higher risk up to 24% was observed for cumulative EQD2 values of > 132 Gy. Considering an EQD2 of 60 Gy for the initial standard chemoradiation, this means that SRS reirradiation doses of 15–16 Gy (EQD2 = 63.7–72 Gy) carry an acceptable risk of radiation necrosis, at least for patients with relatively small recurrent tumor volumes. In a systematic review and analysis of treatment technique of reirradiation for recurrent high-grade gliomas including results of 70 studies with 3302 patients, the median unadjusted rate of brain necrosis after SRS was 8% for patients with a median treatment volume of 10.1 ml and a median dose per fraction of 15 Gy [53]. These values are consistent with those observed in the RTOG study 90–05 that evaluated the risk of radiation necrosis following SRS reirradiation of primary brain tumors and brain metastases with doses of 15–24 Gy [54]. This means in clinical practice that small CTV/PTV margins of 0–2 mm are generally utilized during SRS reirradiation to minimize the risk of brain necrosis, especially when treating larger tumors with cumulative EQD2 exceeding 120 Gy. In contrast, the interval between radiation treatments and the use of concurrent systemic therapies did not emerge as independent factors associated with the development of radiation necrosis in several studies.

### Hypofractionated SRT

Hypofractionated SRT with or without systemic therapy has been frequently used in the setting of recurrent GBM. Treatments include moderately (generally 2.5–3.5 Gy per fraction) and high-dose (5 Gy or more per fraction) hypofractionated schedules (Table 2) [12, 13, 55–69]. Because of its higher degree of precise patient positioning and accurate dose delivery, SRT has superseded conventional RT in clinical practice in the last two decades for the treatment of patients with recurrent tumors. Results of eighteen studies including 976 patients who received stereotactic reirradiation between 2005 and 2020 are shown in Table 2. Using total doses of 30–45 Gy delivered in 2.5–4.0 Gy per fraction, ten studies including 733 patients showed a median overall survival time ranging from 7.5 to 12.5 months [12, 57–59, 65–70]. A similar survival time of 7.3 to 12.5 months has been observed in eight studies including 272 patients who received high-dose hypofractionated SRT at doses of 25–35 Gy in 5–7 Gy per fraction [13, 55, 56, 60–64]. Using a dose of 35 Gy in 10 fractions of 3.5 Gy per fraction, Fogh et al. [58] reported an overall survival time of 11 months in 105 patients with recurrent GBM. A total dose of > 35 Gy resulted in an improved overall survival with no significant differences amongst patients who had chemotherapy and those who did not. Similar survival times of 8 to11 months have been observed in large retrospective multicentric studies using either SRS or hypofractionated SRT [71, 72].

**Table 2:** Hypofractionated stereotactic radiation therapy in recurrent patients with glioblastoma

	Author	Pts	Interval between RT courses	SRT modality	Median dose/fractions	Imaging for planning	Target volume (ml)	CTV/PTV margins (mm)	Systemic therapy (No)	Median PFS (months)	Maedian OS (months)	EQD2 (Gy)	Cumulative EQD2 (Gy)	RN (%)
	Grosu et al., 2005 [13]	34	16	LINAC	30 Gy/6 fr	T1-w ce MRI; 50% MET-PET	15 (1–61)	3 mm	Adjuvant TMZ, 24	NR	8; SRT and TMZ, 11; SRT,6 (p = 0.04)	52.5	112.5	7
	Gutin et al., 2009 [55]	20	15	LINAC	30 Gy/5 fr	T1-w ce, DCE and DSC MRI	34 (2–62)	5 mm	BEV	7.3 (4.4–8.9)	12.5; 54% at 1 year	60	120	0
	Henke et al., 2009 [56]	29	20	LINAC	20 Gy/4–5 fr	T1-w ce MRI, some MET-PET	52.7 (0.9–277)	3–10 mm	TMZ or CCNU, 22	NR	10.2 (2–65)	35	89–94	0
	Fokas et al., 2009 [57]	53	12	LINAC	30–35 Gy/10 fr*	T1-w ce MRI	35 (3–204)	3 mm	ACNU or PVC, 25	9% at 1 year	22% at 1 year	0	91.5–102.1	0
	Fogh et al., 2010 [58]	105	8	LINAC	35 Gy/10 fr*	T1-w ce MRI	14; 33 after surgery	None	TMZ, 26; other CHT, 22	NR	11	48.1	108.1	< 1
	Minniti et al., 2011 [59]	36	14	LINAC	37.5 Gy/15 fr	T1-w ce MRI	32.1 (22.3–72.4)	CTV, 2–3 mm PTV, 2 mm	concomitant TMZ	5; 42% at 6 m	9.7; 33% at 1 year	42.2	102.2	8
	Shapiro et al., 2012 [60]	20	14.7	LINAC	30 Gy/5 fr	T1-w ce MRI	35.3 (2.7–62.1)	5	BEV	7.5	12.2	56.4	116.4	0
	McKenzie et al., 2013 [61]	32	14.2	LINAC	30 Gy/6 fr	T1-w ce MRI	8.5 (0.4–46.5)	2–3 mm	None	NR	8.6; 34% at 1 year	60	120	9
	Minniti et al., 2013 [62]	38	15.5	LINAC	30 Gy/5 fr	T1-w ce MRI; some F-DOPA-PET	30.3; 12.3–53.4	CTV, 2–3 mm PTV, 1–2 mm	daly TMZ	6 24% at 1 year	12.4; 53% at 1 year	60	120	7
	Greenspoon et al., 2014 [63]	31	> 3	LINAC	25–35 Gy/5 fr	T1-w ce MRI	12	1 mm	TMZ	7	9 months	43.75–78.5	103.7–138.5	13
	Minniti et al., 2015 [64]	42	14	LINAC	25 Gy/5 fr	T1-w ce MRI	33.1; 10.3–72.4	CTV, 5 mm; PTV, 1–2 mm	FTM, 23; BEV, 19	+ BEV50% and + FTM18% at 6 m	+ BEV,30%; + FTM, 8.3% at 1 year	43.7	103.7	7
	Youland et al., 2017 [65]	26	20.9	LINAC	35–40 Gy/10 fr	T1-w ce MRI	NR	PTV, 0–2 mm	BEV	2.6	4.9	48.2	108.2	4.3
	Palmer et al., 2018 [66]	87	10.8	LINAC	35 Gy/10 fr*	T1-w ce MRI	35 (18–41.3)	2–5 mm	BEV before or after SRT	NR	11.9 BEV before, 13.9 BEV after SRT	48.2	108.2	NR
	Schernberg et al., 2018 [67]	24	21.9	LINAC	45 Gy/18 fr	T1-w ce MRI	104 (5.5–270)	CTV, 3 mm; PTV, 2 mm	BEV, 22 pts	6.7	10.5	55.6	115.6	0
	Tsien et al., 2019 [68]	170	NR	LINAC	35 Gy/10 fr	T1-w ce MRI	53 (4–411)	3–5 mm	BEV/SRT or BEV alone	54% vs 29% at 6 m (p = 0.001)	BEV + SRT, 10.1 vs BEV, 9.7 (p = 0.5)	48.1	108.1	0
	Chan et al., 2020 [12]	51	21	LINAC	35–40 Gy/15 fr*	T1-w ce MRI + FLAIR images	145.3 (10.6–432)	CTV, 5 mm PTV, 3 mm	BEV	NR	7.5	37.6–46.6	96.7–106.6	4.4
	Kaul et al., 2020 [69]	133	14	LINAC	41.8–49.4/12–15 fr*	T1-w ce MRI	61.9	1–5 mm	TMZ, 58	NR	6	60.6–71.6	120.6–131.6	7.6
	Saeed et al., 2020 [70]	45	20.2	Proton	42.6 Gy/20 fr	T1-w ce MRI	NR	NR	TMZ,16;BEV,4; both,10	13.9 (8.2–20)	14.2 (9.6–16.9)	47.1	107.1	8.8

RT, radiation therapy; CTV, clinical target volume; PTV, planning target volume; PFS, progression-free survival; OS, overall survival; EQD2, equivalent dose normalized to 2 Gy;

LINAC, linear accelerator; T1-w ce MRI, T1-weighted contrast-enhanced magnetic resonance imaging; DCE, dynamic contrast enhanced; DSC, dynamic susceptibility contrast;

*majorty of patients; MET-PET/CT, methionin positron emission tomography/computed tomography; FTM, fotemustine; CCNU, lomustine;TMZ,temozolomide;

BEV, bevacizumab; F-DOPA-PET, 3,4-dihydroxy-6-[18F]-fluoro-l-phenylalanine (F-DOPA)-PET; FLAIR, Fluid-attenuated inversion recovery

Several studies has been evaluated the use of SRT in combination with systemic therapies, as shown in Table 2. In a series of 36 patients with recurrent GBM who received moderately fractionated SRT (37.5 Gy in 15 fractions) and concurrent temozolomide at University of Rome Sapienza, Sant’Andrea Hospital, median overall survival and progression-free survival times were 9.7 months and 5 months, respectively [59]. Using hypofractionated SRT (30 Gy in six fractions) with or without concurrent temozolomide, Grosu et al. [13] observed a median survival time of 11 months after chemoradiation and 6 months after SRT alone (p = 0.04), and similar survival benefits have been reported in other few series using high-dose hypofractionated SRT (25–35 Gy in five fractions) and temozolomide [62, 63]. In all above mentioned studies, longer survival time was associated with the presence of tumors with a methylated enzyme O6-methylguanine-DNA methyltransferase (MGMT) gene promoter.

Survival benefits have been reported following fractionated SRT and concurrent bevacizumab [12, 55, 60, 64–68]. Gutin et al. [55] observed a median overall survival time of 12.5 months and 1-year survival rate of 54% in twenty patients who received 30 Gy in 5 fractions to the recurrent tumor with SRT; median progression-free survival time and 6-month rates were 7.4 months and 65%, respectively. In a small retrospective study comparing hypofractionated SRT (25 Gy in 5-Gy fractions) plus bevacizumab or the alkylating agent fotemustine, median survival times and 12-month survival rates were 11 months and 30% for patients treated with SRT and bevacizumab and 8.3 months and 5% for those treated with SRT and fotemustine (p = 0.01); respective median progression-free survival times were 6 and 4 months (p = 0.01). In a recent multi-institutional, prospective randomized phase II trial (NRG Oncology/Radiation Therapy Oncology Group (RTOG) trial 1205) designed to evaluate the safety and efficacy of reirradiation for recurrent GBM with modern radiation techniques, a similar survival of 10.1 months has been observed following hypofractionated SRT (35 Gy/10 fractions) and concurrent bevacizumab [68].

A risk of radiation necrosis less than 10% is generally reported after SRT for cumulative EQD2 doses less than 120 Gy following either moderately or high-dose fractionated schedules (Table 2). Of note, the risk remains low despite:—higher median treated volumes in the range of 8.5–34 ml and 33–145 ml for high-dose and moderately fractionated radiation schedules, respectively, and—the use of GTV-to-CTV margins up to 5 mm. In the respect of relatively few reported cases, radiation necrosis is usually observed in recurrent tumors that generally receive cumulative EQD2 doses > 120 Gy to volumes > 40 ml [13, 63–65]. Of note, the risk remains low after SRT in combination and concurrent systemic therapy, with a reported lower risk in patients receiving SRT and bevacizumab [12, 64, 68, 73]

### Conventionally fractionated SRT

Several series reported on the efficacy and safety of conventionally fractionated SRT in patients with recurrent gliomas (Table 3) [30, 74–79]. Using a median dose of 36 Gy delivered in 18 fractions of 2 Gy per fraction, the reported median survival time ranges from 6.7 to 11.5 months and progression-free survival time from 2.5 to 5 months. In a large series of 172 patients with recurrent low- and high-grade gliomas treated with a second course of conventionally fractionated SRT (36 Gy in 2-Gy fractions), Combs et al. [74] reported median overall survival and progression-free survival times of 8 and 5 months, respectively, for 59 patients with GBM.

**Table 3:** Conventionally fractionated stereotactic radiation therapy in recurrent  patients with glioblastoma

Author	Pts	Interval between RT courses	RT modality	Median dose/fractions	Imaging for planning	Median target volume (ml)	CTV/PTV margins (mm)	Systemic therapy	Median PFS (months)	Median OS (months)	EQD2 (Gy)	Cumulative EQD2 (Gy)	RN (%)
Combs et al., 2005 [74]	59	10	LINAC	36 Gy/18 fr	T1-w ce MRI	49.3 (2.5–636)	5–10 mm	TMZ, Carmustine, PCV, 36	5 (1–21)	8 23% at 1 year	36	96	0.6
Sholtyssek et al., 2013 [75]	53	13.4	LINAC	36 Gy/18 fr*	T1-w ce MRI	110.4. (1.8– 378)	CTV, 5 mm; PTV, 3–5 mm	TMZ,12; Carbo/etoposide, 24	4.3 33% at 6 m	7.7 24% at 1 year	36	96	0
Flieger et al., 2014 [76]	49	NR	LINAC	36 Gy/18 fr*	T1-w ce MRI	34.9 (1.95–157.9)	5–10 mm	CHT, 38; BEV,18	4.9	7.8	36	96	0
Wick et al., 2014 [77]	91	21	LINAC	36 Gy/18 fr	T1-w ce MRI	size > 2.5 cm in 35 pts	10 mm	SRT + APG101, 58 SRT, 21	2.5. 4.5(+ APG101)	SRT, 11.5; SRT + APG101, 11.5	36	96	1.2
Schnell et al., 2017 [78]	85	18.5	LINAC	36 Gy/18 fr	T1-w ce MRI	128	5–10 mm	Concomitant BEV, 33; Concomitant/ manteinance BEV, 25; BEV + IRI, 27	NR	concomitant BEV, 8; concomitant/ manteinance BEV, 13.1; BEV + IRI, 6.6	36	96	0
Shen et al., 2018 [79]	63	27.6	LINAC	41.4 Gy/23 fr	T1-w ce, DCE, DSC MRI	202 (20–901)	5–10 mm	TMZ, 35; BEV, 5; TMZ/BEV, 6	NR	6.7	41.4	101.4	3.4
Fleischmann et al., 2019 [30]	124	18	LINAC	36 Gy/18 fr*	T1-w ce MRI	118.1 (22.6–385.5)	CTV, 5 mm PTV, 3 mm	BEV, 95	5	9	36–48.4	96–108.4	6.9

LINAC, linear accelerator; CTV, clinical target volume; PTV, planning target volume; PFS, progression-free survival; OS, overall survival; EQD2, equivalent dose normalized to 2 Gy;

*Majorty of patients; SRT, stereotactic radiotherapy; T1-w ce MRI, T1-weighted contrast-enhanced magnetic resonance imaging; DCE, dynamic contrast enhanced; DSC, dynamic susceptibility;

BEV, bevacizumab; TMZ, temozolomide; IRI, irinotecan; Carbo, carboplatin

The superiority of combined chemoradiation versus radiation alone remains to be better defined [30, 76, 78, 79]. Schnell et al. [78] conducted a retrospective three-arm study of 105 patients with recurrent malignant gliomas who were treated with conventionally fractionated SRT (36 Gy in 2-Gy fractions) and concurrent bevacizumab with or without maintenance therapy, or bevacizumab/irinotecan between 2008 and 2014. The authors observed a significantly improved median post-recurrence survival time of 13.1 months for patients receiving reirradiation in combination with concurrent and maintenance bevacizumab compared to a survival time of 8 months for those receiving systemic therapy only or concurrent reirradiation and bevacizumab without maintenance therapy. A recent SRT-specific update of the same study group (n = 161) could not confirm long-term differences according to maintenance therapy; post-recurrence survival time was 9 months in both arms, but toxicity was significantly reduced among bevacizumab patients compared to those receiving reirradiation alone [30].

Using conventionally fractionated SRT, a low risk of radiation necrosis of 0.8% to 6.8% has been observed in six studies including 439 patients with recurrent GBM (Table 3). With a median total dose of 36 Gy delivered in 18 fractions of 2 Gy per fraction (cumulative EQD2 = 96 Gy), the risk remains low even in series with a median target volume of about 100 ml or higher and when using large safety margins up to 10 mm with the aim of including potential microscopic spread.

## Prognostic factors and scores for reirradiation

Several prognostic factors have been correlated with clinical outcomes following reirradiation in patients with GBM. Age at reirradiation, Karnofsky performance status (KPS), tumor grade (grade III versus grade IV glioma) are well recognized prognostic factors associated with longer survival, whereas the role of other factors, including tumor volume, surgery before reirradiation, time interval from first course of RT, use of concurrent systemic therapy, and MGMT promoter methylation remains controversial [43, 54, 56–58, 64, 69, 71, 74, 75, 80–85]. In this regard, some, but not all, studies failed to demonstrate the favorable impact of surgery before reirradiation [57, 80, 82, 84, 93] and time interval between initial standard chemoradiation and reirradiation [56, 58, 64, 75, 81, 82, 85]; however, the majority of authors suggest a minimum interval of six month-interval between the two radiation courses.

Several prognostic scores have been developed to assess the clinical prognosis of patients undergoing reirradiation and to assist with patient selection [71, 75, 79, 85–93]. Using several prognostic factors, including WHO grade, age, gender, MGMT methylation status, time interval between first and second course of RT, and KPS, Niyazi et al. [92] developed a reirradiation risk score to independently predict survival in 565 patients from the German Cancer Consortium (DKTK)/radiation oncology group (ROG) database. Based on multivariate analysis, three prognostic groups were identified based on age, glioma grade and KPS, with longer survival in younger patients with better performance status and a grade III glioma. Another report of DKTK-ROG aimed to validate two different prognostic scores previously generated by Combs et al. [89], which included primary histology, time from primary RT to reirradiation, and age, and by Kessel et al. [90], created by adding values for KPS, irradiated volume, and performed resection. Data demonstrated a significant correlation between both scores and overall survival after reirradiation; however, salvage surgery before reirradiation and the time between radiation courses did not emerge as independent prognostic factors for survival, consistent with results of other studies [56, 57, 64, 75, 80–82, 84, 85, 93]. Overall, prognostic scores incorporating both tumor and patient characteristics are useful to provide recommendations to bear on clinical decisions. In this regard, the impact of other potential prognostic factors, e.g. tumor molecular markers, extent of resection, and concurrent systemic therapies, should be evaluated and validated in large series of irradiated gliomas.

## Conclusions

Reirradiation is an effective and safe treatment in the management of recurrent GBM. For appropriately selected patients, both SRS and SRT, either hypofractionated or conventionally fractionated regimens, are feasible therapeutic options associated with similar median overall survival in the range of 6 to 12 months and relatively low toxicity.

Several studies have investigated the effect of reirradiation in combination with systemic therapy, although its favorable impact on survival remains controversial. Concomitant and/or adjuvant treatment with temozolomide has resulted in longer overall survival and progression-free survival times compared with radiation alone, but this is generally limited to MGMT methylated tumors [13, 48, 63, 72]; in addition, no clear survival advantages have been observed by other authors [58, 74, 85]. Some studies suggested significantly longer survival with the addition of bevacizumab to both SRS and fractionated SRT compared to reirradiation alone [38, 50, 52, 76, 78]; in contrast, other studies failed to demonstrate survival advantages [30, 68, 69, 85]. Overall, the different prognostic impact of chemoradiation over radiation alone in patients with recurrent GBM remains to determined in prospective trials.

Another important question to be solved is the potential superiority of concurrent/adjuvant systemic therapy in combination with reirradiation over systemic treatment alone. In a secondary analysis of NRG Oncology/RTOG trial 0525 evaluating dose-dense versus standard dose temozolomide in newly diagnosed GBM, Shi et al. [94] investigated the effect of reirradiation or systemic treatment after tumor progression in 637 patients who received systemic treatment (44%, bevacizumab for almost all patients), reirradiation alone (4%), combined radiation and systemic therapy (10%), or no treatment (42%). Median survival times were 4.8, 8.2, 10.6, and 12.2 months for patients who received no treatment, radiation treatment only (SRS, fractionated RT or brachytherapy), systemic therapy, or radiation and systemic therapy, respectively. Patients receiving no salvage treatment had significantly lower survival than those receiving radiation alone, systemic therapy or a combination of both; however, survival analysis showed no significant difference among patient groups who received some form of treatment. In the NRG Oncology/RTOG 1205 phase II randomized trial evaluating the efficacy and toxicity of hypofractionated SRT and concurrent bevacizumab versus bevacizumab alone in 182 patients with recurrent GBM, Tsien et al. [68] observed an equivalent median survival time of 10.1 months for patients receiving the combined treatment and 9.7 months for those receiving bevacizumab alone; however, the combined treatment was associated with better 6-month progression free survival (54% vs 29%, p < 0.001). The treatment was well tolerated with few acute (5%) and no delayed grade 3 or more toxicity, confirming the safety of reirradiation using modern RT techniques.

Appropriate patient selection is the key to improve clinical outcome. International guidelines recommend to consider reirradiation for recurrent or progressive GBM in young patients with good performance status, especially after a long period from prior radiation [95, 96]. In this regard, recent prognostic scoring models based on patient and tumor characteristics can be used as guidelines for clinical decision.

The risk of severe radiation-induced complications after reirradiation is a major concern in patients previously exposed to high doses of RT, even with the further optimization obtained with the use of stereotactic techniques. Clinical deterioration due to radiation necrosis has been reported in up to 25% of patients. Reirradiation doses and treatment volumes must both be considered when evaluating the risk of brain necrosis. This risk remains generally low (less than 10%) for cumulative EQD2 doses around 100–110 Gy, but may increase up to 25% for cumulative EQD2 > 130 Gy. Beyond the limit of linear quadratic model when comparing the relative biologic effectiveness of large fraction doses with typical conventional fractionated regimens [29], differences in cumulative EQD2 between the reirradiation techniques may, at least in part, explain the higher risk of brain necrosis after SRS and high-dose hypofractionated SRT versus conventionally fractionated or moderately hypofractionated SRT. This means in clinical practice that SRS or high-dose fractionated SRT would be used for small targets of 5–15 ml, while fractionated SRT using 1.8–2.5 Gy fractions should be preferred for large tumors, particularly if located close to eloquent structures. An advantage of fractionated schedules is the use of larger GTV-to-CTV margins to cover microscopic spread of disease compared with SRS, where the extent of tumor is usually defined by the lesion visible on postcontrast T1-weighted MR imaging; in this regard, the use of PET-CT for tumor definition may improve tumor definition and ensure better coverage of tumor [13–19].

In conclusion, reirradiation has emerged as an effective and safe treatment option for selected patients with recurrent GBM. Using similar biologically equivalent dose, different radiation techniques result in similar survival outcomes. Treatments are associated with a relatively low risk of toxicity when the appropriate radiation technique is carefully chosen on the basis of the size and location of the tumor and in the respect of recommended cumulative dose limits for the brain. Future research includes the use of advanced imaging for tumor definition and the survival impact of different dose-fractionation schedules with or without concomitant/adjuvant systemic therapy. Additionally, studies should report data on tolerance dose of normal brain tissue to reirradiation, as well neurocognitive status and quality of life of patients undergoing the treatment.

## Data Availability

All data supporting the results of this review are published in the cited references.
